# One Day in Denmark: Nationwide point-prevalence survey of human bacterial isolates and comparison of classical and whole-genome sequence-based species identification methods

**DOI:** 10.1371/journal.pone.0261999

**Published:** 2022-02-11

**Authors:** Ana Rita Rebelo, Tobias Ibfelt, Valeria Bortolaia, Pimlapas Leekitcharoenphon, Dennis Schrøder Hansen, Hans Linde Nielsen, Svend Ellermann-Eriksen, Michael Kemp, Bent Løwe Røder, Niels Frimodt-Møller, Turid Snekloth Søndergaard, John Eugenio Coia, Claus Østergaard, Michael Pedersen, Henrik Westh, Frank Møller Aarestrup

**Affiliations:** 1 Technical University of Denmark, National Food Institute, Kongens Lyngby, Denmark; 2 Hvidovre Hospital, Department of Clinical Microbiology, Hvidovre, Denmark; 3 Herlev Hospital, Department of Clinical Microbiology, Herlev, Denmark; 4 Aalborg University Hospital, Department of Clinical Microbiology, Aalborg, Denmark; 5 Aarhus University Hospital, Department of Clinical Microbiology, Aarhus, Denmark; 6 Odense University Hospital, Department of Clinical Microbiology, Odense, Denmark; 7 Slagelse Hospital, Department of Clinical Microbiology, Slagelse, Denmark; 8 Rigshospitalet, Department of Clinical Microbiology, København, Denmark; 9 Hospital of Southern Jutland, Department of Clinical Microbiology, Sønderborg, Denmark; 10 Sydvestjysk Hospital, Department of Clinical Microbiology, Esbjerg, Denmark; 11 Vejle Hospital, Department of Clinical Microbiology, Vejle, Denmark; 12 Department of Clinical Medicine, University of Copenhagen, Copenhagen, Denmark; Universitatsklinikum Hamburg-Eppendorf, GERMANY

## Abstract

**Objectives:**

Implementing whole-genome sequencing (WGS) technologies in clinical microbiology laboratories can increase the amount and quality of information available for healthcare practitioners. In this study, we analysed the applicability of this method and determined the distribution of bacterial species processed in clinical settings in Denmark.

**Methods:**

We performed a point-prevalence study of all bacterial isolates (n = 2,009) processed and reported in the Clinical Microbiology Laboratories in Denmark in one day in January 2018. We compared species identification as performed by classical methods (MALDI-TOF) and by bioinformatics analysis (KmerFinder and rMLST) of WGS (Illumina NextSeq) data. We compared the national point-prevalence of bacterial isolates observed in clinical settings with the research attention given to those same genera in scientific literature.

**Results:**

The most prevalent bacterium was *Escherichia coli* isolated from urine (n = 646), followed by *Staphylococcus* spp. from skin or soft tissues (n = 197). The distribution of bacterial species throughout the country was not homogeneous. We observed concordance of species identification for all methods in 95.7% (n = 1,919) of isolates, furthermore obtaining concordance for 99.7% (n = 1,999) at genus level. The number of scientific publications in the country did not correlate with the number of bacterial isolates of each genera analysed in this study.

**Conclusions:**

WGS technologies have the potential to be applied in clinical settings for routine diagnostics purposes. This study also showed that bioinformatics databases should be continuously improved and results from local point-prevalence surveys should not be applied at national levels without previously determining possible regional variations.

## Introduction

Of the 57 million yearly deaths worldwide, 8.5 million (approximately 15%) are the direct result of infectious diseases [1]. This leads to major expenditures associated with diagnostics, treatment and infection control, as well as health impacts and societal costs associated with illness [2,3]. Out of the multiple potential bacterial infectious agents, current surveillance programs and control efforts are often focused on few pre-defined microorganisms and might not adequately reflect the true prevalence and burden of bacterial diseases [4,5]. In Denmark, large efforts go into tracking, reporting and controlling selected pathogens, namely methicillin-resistant *Staphylococcus aureus* (MRSA), Shiga toxin-producing *Escherichia coli* (STEC), ESBL-producing invasive *E*. *coli*, carbapenemase-producing organisms (CPO), vancomycin-resistant enterococci (VRE), *Neisseria gonorrhoeae* and *Neisseria meningitidis* isolates, amongst others (www.ssi.dk). Similarly, for Europe, the European Centre for Disease Prevention and Control (ECDC) has in place surveillance systems for many of the same pathogens, and the World Health Organization (WHO) includes several antimicrobial-resistant bacterial species of concern in their “WHO priority pathogens list for R&D of new antibiotics” [6]. These systems are focused on bacteria causing infections considered most severe or those considered to have the highest potential to be transmitted or to be refractory to antimicrobial therapy. New surveys and research studies are regularly concerned with novel emerging issues often because of severity of disease, risk of spread of antimicrobial resistance mechanisms or even the emphasis put on novel risks by the media and politicians [7,8]. Globally, a very large number of studies has reported on prevalence and antimicrobial resistance in selected bacterial species and in specific clinical settings. Examples are the SENTRY program which analysed bloodstream infection isolates throughout a 20-year study incorporating over 200 medical centres worldwide [9], and the ECDC reports on the prevalence of healthcare-associated infections in point-prevalence surveys undertaken in 2011 and 2012 [10], and from 2016 to 2017 in the EU/EEA [11]. Surprisingly, very few studies have reported on the relative prevalence of all bacterial species observed nationally without pre-selection of settings or species. One such example corresponds to a five-year analysis of clinical samples at one Ethiopian hospital [12]. These factors might mask the true burden of human bacterial pathogens and we speculated that some bacteria frequently targeted by surveillance efforts can represent a lower healthcare burden than expected, while certain bacterial species might represent a high risk for human health while not being given sufficient attention. To our knowledge, the occurrence of all bacteria observed at the clinical microbiological laboratories in Denmark has not been yet elucidated, although several reports and studies have focused on specific bacterial species, isolates from certain sample sources or on the prevalence of multi-drug resistant bacteria [13–16].

Currently, all clinical microbiology laboratories in Denmark have access to Matrix Assisted Laser Desorption Ionization-Time Of Flight (MALDI-TOF) technologies to identify bacterial pathogens in their daily routine work, although many urine isolates are identified directly from growth on chromogenic agars combined with an easy to perform confirmatory test like the indole spot test. However, the recent improvements and the decrease in cost of next generation sequencing technologies might allow for their application in clinical settings [17]. Implementing whole-genome sequencing (WGS) processes in clinical microbiology laboratories might lead to accurate species identification of bacterial pathogens, while providing information on antimicrobial resistance and virulence genes and allowing data to be processed in the exact same way throughout whole regions or countries. Furthermore, those data can be easily stored and transferred, and can be retrospectively screened if suspicion of an outbreak arises or if new mechanisms of antimicrobial resistance are discovered [18,19]. Although several publications have discussed the potential of employing WGS technologies in clinical settings, to date very few studies effectively analysed the applicability of WGS in a clinical setting, and a more limited number of studies did not focus on pre-selected species [20–23].

To obtain an unbiased overview of bacterial species distribution in clinical settings in Denmark, we conducted the “One Day in Denmark” point prevalence survey including all clinical microbiological laboratories in Denmark and covering all bacterial isolates processed on a single day, from a population of 5.8 million people. We describe our observations regarding the distribution of bacterial species and sample sources and we present a comparison of species identification performed through currently used classical diagnostics methods, in particular MALDI-TOF, and through WGS and different bioinformatics analyses to evaluate the applicability of WGS in a routine clinical context. Furthermore, we compare our findings with estimations of research attention attributed to each bacterial genus.

## Materials and methods

### Bacterial isolates

The study involved all 11 Departments of Clinical Microbiology (DCM) in Denmark including Herlev Hospital, Herlev; Hvidovre Hospital, Hvidovre; Nykøbing F. Sygehus, Nykøbing F; Odense Universitetshospital, Odense; Rigshospitalet, København; Slagelse Sygehus, Slagelse; Sydvestjysk Sygehus, Esbjerg; Sygehus Lillebælt, Vejle; Sygehus Sønderjylland, Sønderborg; Aalborg Universitetshospital, Aalborg; and Aarhus Universitetshospital, Skejby. Geographical provenience of samples was anonymized and each of the 11 DCM received a code (from F1 to F11) for the purpose of this study. The DCM provided all isolates from routine analyses (n = 2,073) present on Wednesday, January 10, 2018, and for which species identification and antimicrobial resistance results had been reported by the responsible clinical microbiologist to the associated hospital or requesting general practitioners. These isolates corresponded to the clinically relevant bacteria recovered and analysed after culturing patients’ biological samples in the DCM, and did not incorporate surveillance nor environmental samples. Metadata including isolate source and species identification were provided. All isolates were anonymized and no patient identifiers were transferred. Isolate source and species identification were normalized for analyses, including correction of species for 13 isolates due to very high probability of mislabelling, which was confirmed by re-analysis through MALDI-TOF in 12 cases (S1 and S2 Tables). The project was approved by local Danish Data Protection Agencies in each Region and Material Transfer Agreements were entered between DTU and all DCM.

The cultures were collected by car and transported to the Technical University of Denmark (DTU) within 24 hours of collection maintaining a refrigeration (agar plates) or freezing (glycerol stocks) temperature. After purity check, and sub-culture for purity when needed, the isolates were stored at -80°C in glycerol stocks. According to the metadata provided, 2,024 isolates were bacteria and 49 were yeasts. Only the bacterial isolates were analysed in this study. Of these, 19 were excluded during further laboratory procedures due to: i) being correctly identified as yeasts (where the metadata reported *Streptococcus agalactiae* (n = 2) and *Streptococcus dysgalactiae* (n = 1)); ii) loss of viability (where the metadata reported *N*. *gonorrhoeae* (n = 5), *Streptococcus pneumoniae* (n = 3), *Campylobacter jejuni* (n = 2) and non-haemolytic *Streptococcus* sp. (n = 1)); and iii) mixed cultures impossible to purify to the level of single species mentioned in the metadata (*Kocuria* sp. (n = 1), *Moraxella catarrhalis* (n = 1), *Proteus hauseri* (n = 1), *Rothia dentocariosa* (n = 1) and *N*. *gonorrhoeae* (n = 1)). Four new isolates of clinical relevance were obtained from mixed cultures, yielding a final collection of 2,009 bacterial isolates with available clinical metadata which were analysed in this study.

### Classical microbiology methods for species identification

Species identification was performed at the DCM using routine methods including MALDI-TOF (Bruker Daltonik, Bremen, Germany) or Vitek MS (BioMerieux, Marcy l’Etoile, France) and/or analysis of specific colony morphology on selective culture plates or chromogenic plates according to each laboratory’s standard operating procedures. Library versions vary between DCM and are continuously updated by manufacturers and supplemented internally and specifically for particular genera such as *Vibrio*, *Yersinia* and *Staphylococcus*.

### Whole genome sequence-based species identification

Genomic DNA was extracted from 2,009 bacterial isolates using the Easy-DNA^TM^ Kit (Invitrogen, Carlsbad, CA, USA) and DNA concentrations were determined using the Qubit^TM^ dsDNA high-sensitivity (HS) and/or broad-range (BR) assay kits (Invitrogen, Carlsbad, CA, USA). Genomic DNA was prepared for Illumina pair-end sequencing using the Illumina (Illumina, Inc., San Diego, CA, USA) NexteraXT® DNA Library Prep Reference Guide (Document #15031942, v03, February 2018) and NextSeq System Denature and Dilute Libraries Guide (Document #15048776, v03, April 2018). The libraries were sequenced using the Illumina NextSeq 500 platform. The raw reads were *de novo* assembled using the Center for Genomic Epidemiology pipeline for assembly and quality control. Quality thresholds were set at maximum 500 contigs per genome and maximum 0.5 million base-pairs of deviation from expected genome size.

Species identification was performed using bioinformatics tools KmerFinder [24,25] which performs k-mer alignment from WGS data (https://cge.cbs.dtu.dk/services/KmerFinder/) and Ribosomal Multilocus Sequence Typing (rMLST) [26] including 53 ribosomal genes using pubmlst database (https://pubmlst.org/rmlst/). Detection of the *mecA* gene was performed using ResFinder [27] 4.0 (https://cge.cbs.dtu.dk/services/ResFinder/) for 347 *S*. *aureus* or *Staphylococcus argenteus* isolates.

Raw sequence data have been submitted to the European Nucleotide Archive (http://www.ebi.ac.uk/ena) under study accession no.: PRJEB37711. A complete list of genomic sequence data is available in the S1 Table.

### Estimation of research focus

To estimate research attention associated with all bacterial genera present in our collection we performed a search using the Web of Science platform (December 2019). We extracted the total number of publications indexed in all databases for the pre-set timespans of i) all years and ii) last five years. We further refined the search criteria by the field “Countries/Regions” and extracted the corresponding number of publications from Denmark. The search was performed both at genera level and species level, and both with and without the inclusion of the keyword “clinical” (S3 Table). To compare the data we first calculated the ratio between the number of isolates in our collection and the number of publications found in the platform. When the resulting proportion was large, it indicated that there were more isolates present in our bacterial collection than what would be expected according to the number of publications. When the resulting proportion was low, the genus was prevalent in the scientific literature but we found few bacterial isolates in our collection. We calculated the mean of these proportions, and compared each of them with the obtained mean: a result above the mean corresponded to genera under-represented in literature and a result below the mean corresponded to genera that were over-represented. In order to obtain the results in a linear scale we converted them using the logarithm of the proportions compared to the mean proportion.

For analyses purposes we used the results obtained for the interval of all time, at national (Danish) level, including the search term “clinical”. To analyse the research focus of one resistance-specific surveillance system, screening of the *mecA* gene through ResFinder 4.0 was used for an estimation of prevalence of MRSA isolates in the clinical collection yielding 11 potential MRSA isolates (S1 Table).

## Results

### Bacterial isolates

The 2,009 isolates were distributed throughout 37 bacterial genera, according to the original data received from the DCM. The most prevalent genus was *Escherichia* (n = 707, 35.1%) followed by *Staphylococcus* (n = 414, 20.6%), *Streptococcus* (n = 232, 11.5%), *Klebsiella* (n = 143, 7.1%), *Enterococcus* (n = 132, 6.6%), *Haemophilus* (n = 62, 3.1%) and *Pseudomonas* (n = 61, 3.0%). Thirty additional genera included 251 (12.5%) isolates (Table 1). Seven isolates were included in the “Undetermined” category (0.3%), which encompassed two isolates identified either as *Aerococcus urinae* or *Escherichia coli*, one isolate belonging either to *Proteus vulgaris* or genus *Enterococcus*, one isolate identified either as *Proteus mirabilis* or Lancefield group B hemolytic *Streptococcus*, one isolate classified as “enterobacteria”, one isolate described as “Gram-positive cocci” and one isolate characterized as “not identified normal flora”. Although the species *Enterobacter aerogenes* was recently reclassified as *Klebsiella aerogenes* [28], and although there have been suggestions to re-evaluate the members of genus *Propionibacterium* (such as renaming *Propionibacterium acnes* as *Cutibacterium acnes*) [29], we maintained the original nomenclature both because the majority of the databases used in this project still contain the previous nomenclature and to be able to perform retrospective literature searches when estimating research attention attributed to each genera throughout “all-years” publications.

**Table 1:** Distribution of the 2,009 clinical isolates according to bacterial genera and sample source according to the metadata provided by the DCM.

Sample source	Urine	Skin or soft tissue	Respiratory system	Blood	Stool or rectum	Eye	Ear	Reproductive system	Abdomen	Undetermined and other sources (1)	Total	Percentage of total
*Escherichia*	646	4	8	23	17				1	8	707	35.2
*Staphylococcus*	25	197	28	40	1	9	12	3	3	96	414	20.6
*Streptococcus*	38	57	23	18	2		1	19	2	72	232	11.5
*Klebsiella*	117	2	7	12			2		2	1	143	7.1
*Enterococcus*	101	4	3	16	1		1	1	3	2	132	6.6
*Haemophilus*			33			10	4			15	62	3.1
*Pseudomonas*	32	13	10			1	1			4	61	3
*Proteus*	48	5	2			1					56	2.8
*Enterobacter*	25		7	2			2		1	3	40	2
*Citrobacter*	28		3					1	3	1	36	1.8
*Aerococcus*	23										23	1.1
*Moraxella*			17			2	1			2	22	1.1
*Propionibacterium*		4		1		1			1	1	8	0.4
*Bacteroides*		1		4						2	7	0.3
*Serratia*			6							1	7	0.3
*Salmonella*	1			3	1						5	0.2
*Stenotrophomonas*			5								5	0.2
*Yersinia*				1	4						5	0.2
*Corynebacterium*		1	1			1				1	4	0.2
*Pasteurella*										4	4	0.2
*Actinomyces*						1				2	3	0.1
*Campylobacter*					3						3	0.1
*Neisseria*	1		1							1	3	0.1
*Raoultella*	3										3	0.1
*Acinetobacter*							1			1	2	0.1
*Morganella*	1	1									2	0.1
*Prevotella*			1							1	2	0.1
*Providencia*	2										2	0.1
Undetermined and other genera (2)	4	1	3	3	3					2	16	0.8
Total	1095	290	158	123	32	26	25	24	16	220	2009	100
Percentage of total	54.5	14.4	7.9	6.1	1.6	1.3	1.2	1.2	0.8	11	100	

(1) Samples of undetermined source (n = 211, 10.5% of the whole collection) or samples originating from sources with few representatives in the collection (Bone or joint (n = 4), Brain or nervous system (n = 3), and Milk (n = 2)).

(2) Isolates of undetermined species (n = 7, 0.3% of the whole collection) or isolates belonging to genera with only one representative in the collection (*Aeromonas*, *Anaerococcus*, *Clostridium*, *Finegoldia*, *Fusobacterium*, *Micrococcus*, *Peptoniphilus*, *Rothia* and *Shigella*).

### Origin of samples

According to the information provided by the DCM, urine cultures was the most prevalent isolate source (n = 1,095, 54.5% of all isolates) followed by skin or soft tissue samples (n = 290, 14.4%), samples originating from the respiratory tract (n = 158, 7.9%) and blood (n = 123, 6.1%). 211 isolates (10.5%) were from undetermined sample sites, including 157 (7.8%) from swabs, 15 from pus (0.7%), 13 from tissues (0.6%), nine from abscess (0.4%), five from secretions (0.2%) and 12 from unknown sources (0.6%). The remaining 132 isolates (6.6%) were distributed among other sources including stool or rectum, eye, ear, reproductive system, abdomen, brain or nervous system, milk, and bone or joint (Table 1).

The most prevalent genus-sample source combination observed in the collection corresponded to *Escherichia* isolates recovered from urine (n = 646), followed by *Staphylococcus* recovered from skin or soft tissue (n = 197), and *Klebsiella* and *Enterococcus* isolated from urine (n = 117 and n = 101, respectively) (Table 1).

The 11 DCM contributed between 41 (2%) and 392 (19.5%) isolates each (S2 Table). For the 12 genera that are represented by more than 10 isolates in the collection (n = 1,928 isolates) their distribution according to the providing DCM presented a few unexpected patterns, with some large deviations observed for certain DCM-species combinations justified by laboratory-specific procedures.

### Whole-genome sequence-based species identification

Three isolates did not respect the quality control parameters’ threshold and were excluded from the WGS analyses (S1 Table).

For 1,872 isolates (93.3%) both KmerFinder and rMLST-based identification were in concordance with the species identification provided by the DCM. This included 48 cases where DCM identification was only performed to genus level and 50 cases where it was performed at Lancefield group level, per DCM protocols. Furthermore, concordance was observed for 1,976 isolates (98.6%) at genus level. After obtaining these results, 91 isolates were reanalysed by MALDI-TOF because of discordance between original identification by the DCM and WGS results. The re-analyses were limited to those isolates for which none of the bioinformatics tool outputs were in concordance with the original species identification. After MALDI-TOF re-testing, the concordance between classical and WGS-based identification was 95.7% (n = 1,919) at the species level and 99.7% (n = 1,999) at the genus level.

Different types of discordance were identified (Table 2). For 70 isolates (3.5%) both bioinformatics tools provided a concordant species identification, which was different from the species identified by the DCM. Of these, discordance at the genus and the species level was observed in 14 (0.7%) and 56 isolates (2.8%), respectively. After re-analysis by MALDI-TOF, the remaining discordances were represented by 28 cases (1.4%), of which 27 (1.3%) corresponded to discordant species identification but concordant genus identification. For two of these 27 cases we were unable to obtain new MALDI-TOF results, as well as for the remaining discordant case which was the only for which a discordance at genus level persisted (Table 2).

**Table 2:** Discordance in species identification performed initially at the DCM, and then by MALDI-TOF re-testing, KmerFinder and rMLST.

Discordance type	Initial DCM species identification	Number of isolates	MALDI-TOF re-test results	KmerFinder species identification	rMLST species identification
Both bioinformatics tools provide the same species identification, which is different from initial DCM identification	*Acinetobacter baumannii*	1	*Acinetobacter pittii*	*Acinetobacter pittii*	*Acinetobacter pittii*
	*Aerococcus urinae*	4	*Aerococcus sanguinicola (n = 2)*	*Aerococcus sanguinicola*	*Aerococcus sanguinicola*
			*Aerococcus urinae (n = 1)*		
			*No peaks found (n = 1)*		
	*Campylobacter jejuni*	1	*Escherichia coli*	*Escherichia coli*	*Escherichia coli*
	*Citrobacter freundii*	2	*Citrobacter braakii*	*Citrobacter braakii*	*Citrobacter braakii*
	*Clostridium difficile*	1	*Bacteroides vulgatus*	*Bacteroides vulgatus*	*Bacteroides vulgatus*
	*Enterobacter cloacae*	1	*Enterobacter aerogenes*	*Enterobacter aerogenes*	*Enterobacter aerogenes*
		1	*Enterobacter bugandensis*	*Enterobacter bugandensis*	*Enterobacter bugandensis*
		8	*Enterobacter cloacae (n = 6)*	*Enterobacter hormaechei*	*Enterobacter hormaechei*
			*Enterobacter xiangfangensis (n = 2)*		
	*Enterococcus faecalis*	1	*Enterococcus faecium*	*Enterococcus faecium*	*Enterococcus faecium*
		1	*Staphylococcus haemolyticus*	*Staphylococcus haemolyticus*	*Staphylococcus haemolyticus*
	*Escherichia coli*	1	*Klebsiella oxytoca*	*Klebsiella michiganensis*	*Klebsiella michiganensis*
	*Haemophilus parainfluenzae*	1	*Moraxella catarrhalis*	*Moraxella catarrhalis*	*Moraxella catarrhalis*
	*Klebsiella oxytoca*	4	*Klebsiella oxytoca*	*Klebsiella michiganensis*	*Klebsiella michiganensis*
	*Klebsiella pneumoniae*	1	*Escherichia coli*	*Escherichia coli*	*Escherichia coli*
		1	*Klebsiella oxytoca*	*Klebsiella oxytoca*	*Klebsiella oxytoca*
		8	*Klebsiella pneumoniae (n = 7)*	*Klebsiella quasipneumoniae*	*Klebsiella quasipneumoniae*
			*Klebsiella variicola (n = 1)*		
		7	*Klebsiella variicola*	*Klebsiella variicola*	*Klebsiella variicola*
	*Neisseria gonorrhoeae*	1	*Aerococcus sanguinicola*	*Aerococcus christensenii*	*Aerococcus christensenii*
	*Pasteurella multocida*	1	*Pasteurella dagmatis*	*Pasteurella dagmatis*	*Pasteurella dagmatis*
	*Pseudomonas stutzeri*	1	*Moraxella catarrhalis*	*Moraxella catarrhalis*	*Moraxella catarrhalis*
	*Raoultella planticola*	1	*Raoultella ornithinolytica*	*Raoultella ornithinolytica*	*Raoultella ornithinolytica*
	*Rothia mucilaginosa*	1	*Haemophilus influenzae*	*Haemophilus influenzae*	*Haemophilus influenzae*
	*Staphylococcus aureus*	1	*No organism ID possible*	*Halomonas hydrothermalis*	*Halomonas hydrothermalis*
		1	*Pseudomonas aeruginosa*	*Pseudomonas aeruginosa*	*Pseudomonas aeruginosa*
		3	*Staphylococcus argenteus*	*Staphylococcus argenteus*	*Staphylococcus argenteus*
	*Staphylococcus hominis*	1	*Staphylococcus saprophyticus*	*Staphylococcus saprophyticus*	*Staphylococcus saprophyticus*
	*Staphylococcus lugdunensis*	1	*Corynebacterium striatum*	*Corynebacterium striatum*	*Corynebacterium striatum*
	*Streptococcus anginosus*	1	*Streptococcus constellatus*	*Streptococcus agalactiae*	*Streptococcus agalactiae*
		1	*Streptococcus constellatus*	*Streptococcus constellatus*	*Streptococcus constellatus*
	*Streptococcus dysgalactiae*	1	*Enterococcus faecalis*	*Enterococcus faecalis*	*Enterococcus faecalis*
	*Streptococcus* sp. Lancefield group B	2	*No organism ID possible (n = 1)*	*Streptococcus dysgalactiae*	*Streptococcus dysgalactiae*
			*Streptococcus canis (n = 1)*		
	*Streptococcus mitis*	1	*Streptococcus oralis*	*Streptococcus oralis*	*Streptococcus oralis*
	*Streptococcus pneumoniae*	2	*Streptococcus mitis*	*Streptococcus mitis*	*Streptococcus mitis*
		2	*Streptococcus pseudopneumoniae*	*Streptococcus pseudopneumoniae*	*Streptococcus pseudopneumoniae*
	*Streptococcus pyogenes*	1	*Corynebacterium striatum*	*Corynebacterium striatum*	*Corynebacterium striatum*
	*Streptococcus sanguinis*	3	*Streptococcus gordonii*	*Streptococcus gordonii*	*Streptococcus gordonii*
One bioinformatics tool in concordance with DCM identification and the other tool discordant	*Citrobacter freundii*	3	Not re-tested	*Citrobacter freundii*	*Citrobacter portucalensis*
	*Enterobacter cloacae*	14	Not re-tested*	*Enterobacter cloacae*	*Enterobacter hormaechei*
		1	Not re-tested		*Enterobacter kobei*
		3	Not re-tested		*Enterobacter ludwigii*
	*Klebsiella oxytoca*	5	Not re-tested	*Klebsiella oxytoca*	*Klebsiella grimontii*
		3	Not re-tested		*Klebsiella michiganensis*
	*Proteus vulgaris*	1	Not re-tested	*Proteus vulgaris*	*Proteus cibarius*
		1	Not re-tested		*Proteus* genomosp. 4
		1	Not re-tested		*Proteus* genomosp. 6
	*Enterococcus casseliflavus*	1	Not re-tested	*Enterococcus gallinarum*	*Enterococcus casseliflavus*
	*Enterococcus gallinarum*	1	Not re-tested	*Enterococcus* sp.	*Enterococcus gallinarum*
	*Escherichia coli*	2	Not re-tested	*Escherichia marmotae*	*Escherichia coli*
	*Pasteurella canis*	1	Not re-tested	*Pasteurella dagmatis*	*Pasteurella canis*
	*Prevotella baroniae*	1	Not re-tested	*Prevotella denticola*	*Prevotella baroniae*
	*Shigella sonnei*	1	Not re-tested	*Escherichia coli*	*Shigella sonnei*
	*Peptoniphilus harei*	1	Not re-tested	*Anaerococcus* sp.	*Peptoniphilus harei*
No agreement between species identification	*Anaerococcus* sp.	1	Not re-tested	*Anaerococcus* sp.	*Anaerococcus lactolyticus*
	*Corynebacterium macginleyi*	1	No organism ID possible	*Corynebacterium simulans*	*Corynebacterium accolens*
	*Corynebacterium tuberculostearicum*	2	No organism ID possible	*Corynebacterium striatum*	*Corynebacterium pseudogenitalium*
	*Enterobacter asburiae*	1	*Enterobacter cloacae*	*Enterobacter cloacae*	*Enterobacter roggenkampii*
	*Enterobacter cloacae*	1	*Enterobacter xiangfangensis*	*Enterobacter xiangfangensis*	*Enterobacter hormaechei*
	*Escherichia coli*	1	*Acinetobacter baumannii*	*Acinetobacter calcoaceticus*	*Acinetobacter baumannii*
	*Klebsiella oxytoca*	1	*Raoultella planticola*	*Raoultella planticola*	*Raoultella ornithinolytica*
	*Neisseria gonorrhoeae*	1	*Actinomyces odontolyticus*	*Streptococcus equinus*	*Granulicatella adiacens*
	*Pasteurella* sp.	1	Not re-tested	*Pasteurella multocida*	*Pasteurella* sp.
	*Prevotella* sp.	1	*Anaerococcus hydrogenalis*	*Anaerococcus sp*.	*Anaerococcus hydrogenalis*
	*Propionibacterium* sp.	1	Not re-tested	*Propionimicrobium* sp.	*Propionimicrobium lymphophilum*
	*Proteus hauseri*	1	*Proteus vulgaris*	*Proteus vulgaris*	*Proteus* genomosp. 4
	*Pseudomonas aeruginosa*	1	*Pseudomonas* sp.	*Pseudomonas alkylphenolica*	*Pseudomonas* sp.
	*Raoultella planticola*	1	*Klebsiella oxytoca*	*Klebsiella oxytoca*	*Klebsiella grimontii*
	*Streptococcus* sp.	1	*Streptococcus mitis*	*Streptococcus mitis*	*Streptococcus infantis*
Undetermined	*Actinomyces turicensis*	1	No peaks found	No output	*Actinomyces turicensis*
	Undetermined	1	*Enterococcus faecium*	*Enterococcus faecium*	*Enterococcus faecium*
		1	*Erwinia persicina*	*Erwinia persicina*	*Erwinia persicina*
		1	*Escherichia coli*	*Escherichia coli*	*Escherichia coli*
		1	*Morganella morganii*	*Morganella morganii*	*Morganella morganii*
		1	*Pseudomonas aeruginosa*	*Pseudomonas aeruginosa*	*Pseudomonas aeruginosa*
		1	*Actinotignum sanguinis*	*Actinotignum schaalii*	*Actinotignum timonense*
		1	No organism ID possible	*Luteipulveratus mongoliensis*	*Barrientosiimonas humi*

*One of these isolates was re-tested using MALDI-TOF for mislabelling verification. The species *E*. *cloacae* was confirmed.

Cells highlighted in orange show discordances that persist after MALDI-TOF re-testing or inability to obtain species identification by the tool(s). Cells highlighted in blue show discordances for isolates not subjected to MALDI-TOF re-testing or inability to obtain species identification by the tool(s).

For 40 isolates (2%), there was concordance between species identification obtained by one bioinformatics tool and the routine method at the DCM, but the other bioinformatics tool provided a different result. Of these, 32 (1.6%) isolates corresponded to discordance using rMLST which all occurred at species but none at genus level. Eight cases were attributed to discordance using KmerFinder (0.4%), with only two of those cases (0.1%) corresponding to misidentification at the genus level.

For 16 isolates (0.8%) it was not possible to obtain an agreement in species identification by any of the three methods, although for 10 (0.5%) isolates the methods were concordant at genus level. After MALDI-TOF re-testing, conventional method and KmerFinder provided concordant species identification for six of the 16 isolates (0.3%), and for these isolates rMLST results were also concordant at the genus level. Furthermore two (0.1%) cases revealed concordance at species level between MALDI-TOF results and rMLST, also agreeing at genus level with KmerFinder. Of the remaining eight discordant cases, six were discordant only at species level; new MALDI-TOF analyses were either unable to characterize those samples to species or even genus level (n = 1 and n = 3, respectively) or the isolates were not re-tested (n = 2). The other two cases correspond to a complete discordance between the three sets of data, of which only one was re-tested through MALDI-TOF maintaining the discrepancy.

Eight cases (0.4%) were classified as undetermined regarding species concordance. For five cases (0.2%) no definite species identification was received from the DCM but results from both bioinformatics tools were in agreement at species level, and MALDI-TOF re-testing results revealed complete species concordance across the species identification methods. In another case (0.05%), the bioinformatics tools provided identification of congeneric species whereas DCM identification was not known. New MALDI-TOF results revealed a different but also congeneric species. An additional case (0.05%) corresponded to inability of KmerFinder to perform species identification, whereas both the DCM method and rMLST identified the same species. MALDI-TOF repetition of this isolate was unable to provide an identification. In the remaining case (0.05%), there was no agreement at the genus level by any of the methods and new MALDI-TOF experiments were unable to characterize the isolate.

The quality of bioinformatics tool outputs varied. KmerFinder identified 1,771 isolates (88.3%) with query genome coverages higher than 90%, 140 isolates (7%) with coverages between 80% and 90%, 19 isolates (0.9%) with coverages between 70% and 80% and 19 isolates (0.9%) between 60% and 70%. Seven isolates (0.3%) were identified with 50% to 60% and an additional seven isolates were identified with 40% to 50% of query coverage. The remaining 43 (2.1%) were identified with coverages lower than 40%. rMLST identified 1,858 isolates (92.6%) with 100% of support, while identifying 114 isolates (5.7%) with support between 95% and 99%. The remaining 34 isolates (1.7%) were identified with less than 95% of support. A full description of the quality outputs and their relationship to species identification concordances and discordances can be found in the S4 Table.

### Estimation of research focus

**Fig 1:**
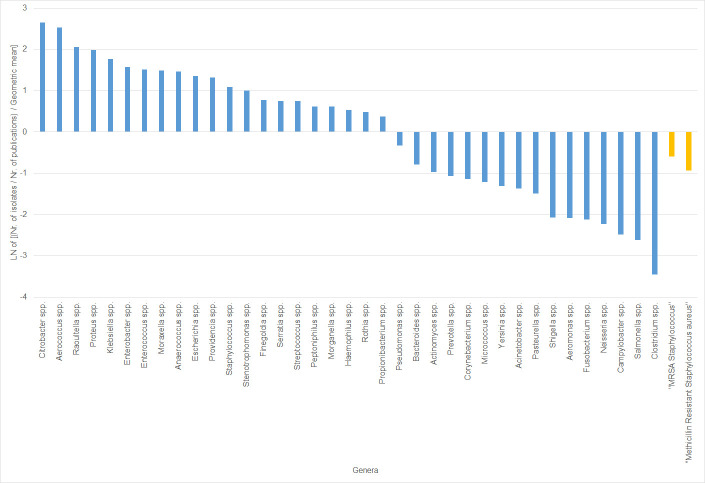
Comparison of prevalence and research attention focus according to bacterial genera.

Minor differences were observed when comparing the ratios calculated with the number of publications worldwide and with the number of publications in Denmark, while comparing the numbers of publications in the last five years and for all time, and with the inclusion of the key-word “clinical” in the search terms (S3 Table). For 21 genera present in the collection it appeared that the number of isolates observed was disproportionally higher than the number of publications focusing on that genus (Fig 1). This difference was particularly notable for the genera *Citrobacter*, *Aerococcus*, *Raoultella*, *Proteus* and *Klebsiella*. Of the 21 genera, nine are included in national surveillance systems, ECDC surveillance systems and/or the WHO priority pathogens list for R&D of new antibiotics (S5 Table). Conversely, for 16 genera it appeared that the number of isolates present in the collection was lower than expected when compared with the number of publications, used as a measure of research focus. Of these, ten are included in national, ECDC and/or WHO lists of priority pathogens or pathogens under surveillance systems. The genera for which this difference was more accentuated were *Clostridium*, *Salmonella*, *Campylobacter*, *Neisseria* and *Fusobacterium*. Of note, the ratio for *Neisseria* genus (-2.22) could have been less accentuated if six additional presumptive *N*. *gonorrhoeae* isolates had not been excluded. Regarding MRSA isolates results seem to indicate that the number of publications focusing on this pathogen is higher than expected when compared with the number of potential MRSA isolates in national clinical settings.

Logarithms of the proportions “number of bacterial isolates of each genera in the collection, per number of publications concerning those genera”, relative to the mean number of bacterial isolates per publications. Results for “all time”, at national (Danish) level, including the search term “clinical”. Positive numbers correspond to genera over-represented in the bacterial collection and negative numbers correspond to under-represented genera. Yellow bars correspond to search terms not corresponding to bacterial genera but instead antimicrobial-resistant bacteria. Number of publications extracted from Web of Science All Databases collection. December 2019.

## Discussion

In this study we presented a point-prevalence survey of all bacterial isolates processed in one day in all clinical microbiology departments across Denmark, and thus encompassing isolates from hospitals and clinical practices. We clearly observe that *E*. *coli* was the predominant bacterial species identified, followed by *S*. *aureus*, *Streptococcus* spp. and *Klebsiella* spp. isolates. Urinary tract infections had the highest prevalence, likely because community samples were also included in this study. Skin and soft tissue infections, respiratory infections and isolates originating from blood culture also represented a high percentage of the observed bacteria. We observed a heterogeneous relation between sample sources and bacterial species, with the most notable exceptions being skin or soft tissue infections mainly associated with *Staphylococcus* and *Streptococcus* genera, stool samples primarily related with *E*. *coli* isolates, eye samples associated with *Staphylococcus* spp. and *Haemophilus* spp. isolates, ear samples related with *Staphylococcus* spp. isolates and reproductive system samples mainly connected with *Streptococcus* spp. isolates. It should be noted that the use of point-prevalence data obtained from only one single day is likely not representative of the seasonal or annual distribution of bacterial species; examples of genera known to be subjected to marked seasonal variations are *Campylobacter* and *Salmonella* which present a much higher national prevalence during summer months (www.ssi.dk). Further work to increase the confidence of point-prevalence results of bacterial isolates in Denmark can include new surveys performed in different seasons or collection of epidemiological data from governmental databases. Our findings corroborate previous research which was however focused on aetiology of hospital-associated infections only. The SENTRY program analyzed bloodstream infection isolates throughout a 20-year study incorporating over 200 medical centers worldwide and showed that the most common etiological agent observed was *S*. *aureus*, followed by *E*. *coli*, *Klebsiella pneumoniae*, *Pseudomonas aeruginosa*, *Enterococcus faecalis*, *Staphylococcus epidermidis*, *Enterobacter cloacae*, *Streptococcus pneumoniae*, *Enterococcus faecium*, and *Acinetobacter baumannii-Acinetobacter calcoaceticus* species complex [9]. Additionally, the ECDC report on the prevalence of healthcare-associated infections revealed a somewhat similar profile of causative agents in hospital associated infections in two point-prevalence surveys from 2016 to 2017 in the EU/EEA, with the most frequently isolated bacterial species being *E*. *coli*, followed by *S*. *aureus*, *Klebsiella* spp., *Enterococcus* spp., *P*. *aeruginosa*, *Clostridium difficile*, coagulase negative staphylococci, *Enterobacter* spp. and *Proteus* spp. [11]. The most prevalent types of infection were respiratory tract infections, urinary tract infections, surgical site infections, bloodstream infections and gastro-intestinal infections, corroborating observations already established in previous surveys undertaken in 2011 and 2012 [10]. In long-term healthcare facilities skin infections also proved prevalent. One study reporting on the relative prevalence of all bacterial species observed nationwide corresponds to a five-year analysis of clinical samples at one Ethiopian hospital, revealing that *S*. *aureus* was the most prevalent bacterial species, followed by *Salmonella* spp., *E*. *coli*, *P*. *aeruginosa*, *Shigella* spp., *K*. *pneumoniae* and *Streptococcus pyogenes*, with other species presenting lower prevalence. The main infection sources were stool cultures, urine samples, ear discharges, wound swabs and blood [12]. This distinct profile at its respective setting indicates that geographical location, and potentially national health-care resources, can strongly affect the epidemiological findings for each particular country.

We observed a moderate degree of homogeneity across the whole country of Denmark, with few exceptions. F3 provided very high percentages of *Haemophilus* spp. (34%), *Aerococcus* spp. (48%) and *Moraxella* spp. (36%) isolates while being responsible for only 10.6% of all isolates collected. These higher numbers of isolates are believed to be due to regional variation in the testing and reporting practices. F4 –responsible for 17.5% of the whole collection–provided 34% and 38% of *Klebsiella* spp. and *Proteus* spp. isolates, respectively, and 28% of all *Escherichia* spp. isolates. Conversely, it only contributed 0.4% (n = 1) of *Streptococcus* spp. isolates, and 0% of all *Aerococcus* spp. and *Moraxella* spp. DCM F4 does not usually perform antimicrobial susceptibility testing for *Streptococcus* spp., *Staphylococcus* spp., *Aerococcus* spp. and *Moraxella* spp. isolates, hence the absence of these genera from the samples processed there. The higher number of isolates belonging to other bacterial genera observed in that laboratory can be attributed to the presence of isolates from the previous day than that of the plate collection, which can be considered as a limitation regarding the point-prevalence results reported here. F7 contributed 1% and 0% of *Escherichia* spp. and *Aerococcus* spp. isolates, respectively, but 5% of the overall collection. F7 does not perform analyses of samples originating from general clinical practice, which likely explains the low prevalence of UTI-associated genera in their laboratory. We expect these findings to be useful both in guiding further research efforts and in making future institutional or governmental decisions. It is important to note that Denmark is a small northern-European country and these observations are likely very dependent on development stage and implementation of clinical microbiology, population size, geographical location and even seasonal variability. Furthermore, we believe that these results demonstrate that regional results of point-prevalence, prevalence or incidence studies should not be applied to national contexts unless properly validated, given the clear variation of species distribution in each laboratory.

Our study showed that there was very high concordance between WGS and the current routine methods for species identification on a collection of bacterial species that were not *a priori* selected. This indicates that WGS has the potential to be routinely used in clinical diagnostics. Currently, MALDI-TOF is the main method used routinely for species identification at DCM in Denmark, but despite its widespread use it has known weaknesses, such as several genera can only be identified at species group or complex level, as in the case of the *Streptococcus mitis* group or the *Enterobacter cloacae* complex. Although MALDI-TOF libraries can be improved by adding own mass spectrum entries as shown in this work for *Staphylococcus argenteus* (Table 2), bioinformatics libraries have the advantage of being transversal to several different sequencing platforms thus allowing for their application in various settings. KmerFinder performed very well but there were limitations in the percentages of query coverage, which were nevertheless above 80% for 95% of cases. Lower coverages were not surprising and are explainable according to the mechanisms of the alignment program, as it performs alignment against full reference genomes. In practice, low percentages of query coverages will be observed for species which present few publicly available reference genomes, few complete genomes or genomes with low quality, or for species which present a naturally high genomic plasticity or variability. rMLST performed better than KmerFinder in terms of confidence as it systematically returned higher percentages of support, mainly since it considers a limited number of conserved genes (approximately 53 genes) for species identification. These are used to calculate a percentage of support corresponding to the percentage of detected species-specific exact-matching alleles in regard to the total number of alleles that support any reported taxa found in the genome (including any unknown taxa). However, rMLST seemed to lead to a slightly higher number of discordances in species identification when compared to KmerFinder, notably for isolates belonging to *Enterobacter* genus (n = 18, or 56.3% of the 32 isolates that rMLST could not identify as the same species as the DCM and KmerFinder). We obtained concordance at genus identification level for all tools in 1,999 isolates (99.7%). In a clinical context identification at this level might prove sufficient as often intrinsic and acquired resistance mechanisms are similar for the different congeneric species, meaning that empiric therapeutic approaches and control efforts might not change even if misidentifying congeneric species. Furthermore, additional information provided by bioinformatics pipelines will complement this identification and allow for more directed clinical and/or therapeutic interventions, such as *in silico* antibiograms [30]. We only observed discordances at genera level in five cases (0.2%), and two cases of undetermined concordance (0.1%) due to absence of either DCM (n = 1) or KmerFinder (n = 1) identifications. These situations might correspond to true failures of one or more of the methods tested or can also be the results of bacterial contaminations or undetected mislabelling which would lead to an incorrect bacterial isolate being analysed. Our findings expand on previous research focused on selected bacterial species. Long et al., 2013 analysed 130 clinical samples through BLAST alignment against the NCBI Nucleotide database, being unable to identify only 13 samples (10%) from the WGS datasets [20]. Similarly, Roach et al., 2015 performed genomic analysis of 1,229 bacterial isolates collected over one year at an intensive care unit; definitive genera identification through classical microbiological diagnostics methods was achieved for 1,054 samples, with 70 of these cases (6.6%) yielding discordances at genus identification level between the laboratory classification and the genomic identification [21]. Hasman et al., 2014 analysed the potential of applying WGS methods directly to 19 urine samples; while the metagenomics analyses yielded complex results, WGS and bioinformatics analyses of 18 of the purified isolates identified those as belonging to the same genera as predicted through conventional identification methods [22]. Likewise, Schmidt et al., 2017 were able to effectively identify the species of six human pathogens from urine samples using WGS and bioinformatics tools, both at biological sample and cultivated bacteria levels [23].

Our comparison between the point prevalence of different pathogens and the research focus suggests that currently there is a discrepancy between the prevalence of some bacteria and research focus. We believe that this is a highly important point and suggest that public health attention as well as funding for control programs and basic research should take into consideration the current prevalence of the pathogens, as an addition to other relevant metrics such as historical epidemiological data from surveillance programs, antimicrobial resistance emergence trends and national and international burdens of disease. It should be noted, however, that the number of research publications is not a flawless representation of research attention paid to certain species, as the number of publications can be high due to the infectiveness of that species or due to severe health impact of the infection, and possibly not directly related to funding attributed to the control of those infections. Additionally, a lower prevalence of certain species highly targeted in scientific research might be a reflection of seasonal variation in prevalence or even demonstrate that the surveillance and research efforts were successful in reducing the burden of that pathogen. Other limitations of the method used to calculate the research focus are a difficulty in estimating the total research focus attributed to bacterial infections and the possibility that some species might be represented in other publications not considered in this study due to those being indexed with broader terms such as “*Enterobacteriaceae”*. Furthermore, many surveillance systems focus on isolates with particular antimicrobial resistance (AMR) profiles. As we did not investigate all AMR determinants it was difficult to estimate the adequacy of such systems, with the example of MRSA having been the only one analysed. It can be noted that a discrepancy in a national context was observed for this particular pathogen, which appeared to be the focus of a high number of research publications and is known to be the main focus of two national research groups while having a low prevalence in the bacterial collection. This difference highlights the success of national surveillance programs and control efforts.

MALDI-TOF is a fast and accurate identification method with very low analysis cost per sample and the potential to be improved, and will likely remain in use throughout clinical microbiology laboratories. However, it is plausible that in the future WGS could be the main diagnostics tools used in clinical microbiology settings, particularly integrating the advance of bioinformatics algorithms and databases and the increase of validation studies performed. User-friendly interfaces that perform complete bioinformatics analyses of genomes are available making this technology functional for healthcare workers that have no previous bioinformatics experience. These data can be stored and shared, and analyses can be performed in the exact same way across different healthcare settings in a reproducible way that registers all analyses parameters and allows for retrospective investigations. Other possible and important applications of this technology in these settings include antimicrobial susceptibility profiling, determination of sequence types or serotypes and timely outbreak detection and control, which are currently not achievable by one single diagnostics technology in place in the DCM. Furthermore, the advance of metagenomics sequencing techniques might in the future allow for application of these bioinformatics methods directly in biological samples.

In conclusion, we present a point-prevalence analysis of all bacterial isolates processed in Denmark in one day from clinical microbiology settings. We compared classical microbiology diagnostics methods (MALDI-TOF) with Illumina NextSeq technology and two bioinformatics tools (KmerFinder and rMLST) and our results show that WGS can be applied in a clinical setting for species identification purposes. We have compared the research attention given to bacterial genera and their effective national prevalence and suggest that regional data should not be applied to national contexts without a previous analysis of potential biases or local variations.

## Supporting information

S1 Fig Comparison of different publication metrics extracted from Web of Science, December 2019, and relation to the point-prevalence of bacterial isolates. (XLSX)

S1 Table Bacterial isolates collected in the “One Day in Denmark” collection and whole-genome sequence quality parameters. (XLSX)

S2 Table Distribution of bacterial isolates according to their genera and the providing DCM, in absolute frequency and in percentages relative to the total number of isolates of those genera. (XLSX)

S3 Table Publication metrics extracted from Web of Science, "All Databases", December 2019 and respective analysis. (XLSX)

S4 Table Quality of KmerFinder and rMLST outputs and description of species identification concordances and discordances. (XLSX)

S5 Table Comparison of the prevalence of bacterial genera studied in this project and their presence or absence from two surveillance systems (SSI at national level and ECDC at international level) and the WHO priority list for research and development. (XLSX)
